# Supplementary material for: Are carbon emissions trading and green financial instruments synergistic? -Comprehensive quantitative research based on content analysis
Source: PLoS One. 2024 Mar 7;19(3):e0298601. doi: 10.1371/journal.pone.0298601 (PMC10919601; doi:10.1371/journal.pone.0298601)
Supplement: S1 Data — (ZIP) [file pone.0298601.s001.zip › Data/地级市Z_F工作报告.zip!/║■─╧/╗│╗»╩╨/└┤╫╘╣1⁄2╓┌║┼í╛┬φ┐╦╩2╛▌═°í┐_2018.docx]

各位代表：

现在，我代表市人民政府向大会报告工作，请予审查，并请各位政协委员和其他列席人员提出意见。

一、2017年工作回顾

刚刚过去的一年，我们坚持以习近平新时代中国特色社会主义思想为指导，全面贯彻党的十八大、十八届三中、四中、五中、六中全会和十九大精神，在省委省政府和市委的坚强领导下，在市人大、市政协的监督支持下，紧紧围绕“一极两带”和“一个中心、四个怀化”战略目标，坚持稳中求进工作总基调，迎难而上，锐意创新，较好地完成了各项目标任务。实现地区生产总值1546亿元（预计数，下同），增长8%；全社会固定资产投资1235.5亿元，增长14.2%；规模工业增加值增长7%；一般公共预算收入136.53亿元，增长11%；社会消费品零售总额620.9亿元，增长10.5%；城、乡居民人均可支配收入分别达24490元、8840元，增长8.6%和11.1%。

经济质量稳步提升。坚定不移贯彻新发展理念，切实把握经济新常态，突出产业发展这个重点，产业及园区发展取得新成效，经济结构更趋合理，发展活力不断增强。工业经济提速升级。产业及产业园区三年倍增计划全面完成，园区标准化厂房面积、招商引资、固定资产投资、规模工业增加值、技工贸收入、税收分别是2014年的5.4倍、2.1倍、2.4倍、2倍、2.5倍、2.1倍，怀化高新区创建国家高新区有望得到国务院审批。沅陵奇力新电子、辰溪润沅新材料、新晃新能源汽车、洪江市沃世达智能制造等一批新兴产业项目投产达效。规模工业利润增长53%，高出全省平均水平33个百分点。农业产业提质增效。新增国家级、省级现代农业产业园20个、市级龙头企业28家，完成农产品加工销售收入376亿元、增长16%。实施优质农产品品牌战略，黔阳冰糖橙荣获中国百强农产品区域公用品牌，碣滩茶荣获中国优秀茶叶区域公用品牌，靖州荣获国家级出口食品农产品质量安全示范区和国家现代农业产业园，会同魔芋、新晃侗藏红米、雪峰山鱼腥草荣获国家地理标志保护产品。第三产业提档加速。入列全国农产品冷链流通标准化试点示范城市。跨境商品交易中心投入使用，华美立家武陵国际商贸城、武陵山现代商贸物流城、义乌中国小商品城等一批重大项目进展顺利。辰溪、通道、新晃三县入列全国电子商务进农村综合示范县。启动创建5A级景区1个、4A级景区3个。靖州、鹤城、沅陵、溆浦、洪江区入列全省精品旅游线路重点县。全年接待游客5000万人次，实现旅游收入350亿元，均增长20%。财税质量不断改善。一般公共预算收入增幅高于全省平均水平3个百分点，连续三年高于GDP增速。全市地方收入非税占比由上年的42.1%下降到35.98%。金融支持实体经济信贷总额达1015亿元、新增100亿元。改革创新活力凸显。坚持以供给侧结构性改革为主线，扎实推进“三去一降一补”，依法关闭煤矿9家、砖厂135家、国有僵尸企业3家，淘汰过剩钢铁产能30万吨，市本级消化商品房库存130万平方米，为实体经济减负5.4亿元。“四跟四走”产业扶贫、“互联网+监督”、“三级一体”警务改革获省委省政府首届管理创新奖，“放管服”改革、集体林业综合改革经验在全国推介，商事制度改革成效显著，信访一体化改革深入实施，市属国有企业实现集中统一监管全覆盖。国家和省级科技创新平台增至34个，高新技术企业达79家，高新技术产业增加值占GDP比重5%。全社会研发经费支出12.5亿元，占GDP比重0.83%，发明专利申请量增速居全省前列。对外开放步伐加快。铁路口岸即将投入使用，落户怀化的500强企业增至29家，进出口企业增至47家，成立香港怀化联谊会。全年招商引资实际到位资金600亿元、增长42%，实际利用外资5041万美元、增长19%，完成进出口总额9500万美元、增长47.9%。

脱贫攻坚扎实推进。坚定不移把脱贫攻坚作为首要政治任务和第一民生工程，突出易地扶贫搬迁和产业扶贫两大抓手，精准脱贫取得阶段性成效。年度任务全面完成。实现17.68万贫困人口脱贫，336个贫困村出列，中方县、洪江市、鹤城区脱贫摘帽已接受省级评估检查，中方县即将接受国家评估检查。“五个一批”纵深推进。实施产业扶贫项目3800余个，建成村级扶贫产业园419个，总结推广沅陵“扶贫车间”试点经验和麻阳“连村联创、抱团攻坚”成功做法。“万企帮万村”深入推进，建立劳务协作基地27个，新增贫困劳动力转移就业1.49万人。完成易地扶贫搬迁5.8万人，超省定任务1.15万人。发放生态补偿资金3524万元，新增生态管护员1.1万人。基本医疗、大病保险实现全覆盖，惠及贫困群众87.07万人，扶贫特惠保覆盖贫困人口72.7万人。深度扶贫精准发力。新增的脱贫攻坚资金和项目，向131个深度贫困村、12278户深度贫困家庭倾斜；完成贫困村农村公路提质改造713.34公里、农网改造升级368个、农村危房改造1.85万户，建设农村综合服务平台1853个；解决农村安全饮水74.58万人，其中贫困人口28.86万人。

城乡面貌不断改观。坚定不移推进城乡协调发展，突出中心城区和县城建设两大重点，成功创建省级文明城市，新型城镇化和美丽乡村建设步伐不断加快。中心城区提质稳步推进。怀化综合客运枢纽站基本建成，城东组团路网基础设施和怀芷路、舞水路改造等项目建设达到预期，新一轮太平溪和舞水河综合治理、湖天大桥改扩建、城市公共停车场等项目加快推进，太平桥片区、人民西路暨鹤州路片区和城中村棚户区改造步伐加快，城区中心市场、迎丰市场、城东市场提质改造顺利实施。56个重点楼盘、重点地段、重点楼体亮化项目全面完成。城区道路交通、噪声扬尘等实现智能监管，环卫保洁、园林绿化、户外广告实行市场化运作，市容市貌更加清新整洁。交通枢纽地位得到巩固。怀邵衡铁路、渝怀铁路增建二线、张吉怀高铁、铁路编组站建设进展顺利，包茂高速怀化至桂林全线贯通，武靖、怀芷高速建设稳步推进，怀芷快速干线加快建设，成功入列“全国性综合交通枢纽城市”。城镇建设协调推进。通道、芷江成功创建国家卫生县城，洪江市托口镇、溆浦县北斗溪镇获评湖南省美丽乡镇，通道县县溪镇入列湖南历史文化名镇。美丽乡村建设加快。完成示范创建项目215个，新增中国少数民族特色村寨14个、中国传统村落32个、经典文化村镇17个。

环境整治成效明显。坚定不移贯彻绿色发展理念，抓住中央环保督察这个契机，着力解决环境突出问题，绿色怀化建设迈出新步伐。中央环保督察组交办件办结率100%。持续推进大气污染防治行动，城区空气优良率达90.1%，环境空气质量综合指数排名全省第2。全面推行河长制，取缔水源污染企业41家，完成畜禽养殖污染治理项目198个，14个集中式饮用水源地水质达标率100%。完成土壤和重金属污染治理项目10个、农村环境综合整治工程341个。突出裸露山体治理，完成造林绿化25万亩，环城生态圈封山育林18.3万亩。新增麻阳锦江、中方舞水国家湿地公园和溆浦国家森林公园，国家级湿地公园、森林公园和自然保护区达16处。

人民生活持续改善。坚定不移贯彻以人民为中心的发展思想，突出增强人民群众获得感这个目标，省市32项重点民生实事全面完成，全面小康实现程度达89%。民生事业加快发展。财政民生支出占一般公共预算支出比重达79.66%。城区教育发展第二个五年行动计划全面完成，新增义务教育学位1.92万个，成功引进长郡、雅礼、衡水3家优质教育资源入驻，“大班额”问题得到一定程度缓解。积极推行城乡义务教育一体化，建成义务教育标准化教学点222个。全市职业学校毕业1.75万人，就业率达99%。新增城镇就业3.6万人，城镇登记失业率为4.23%。新增农村劳动力转移就业3.88万人，大学生创业园建成开园，全市创业带动城乡就业2.96万人。社会保险覆盖率达96%，农村低保标准提高至3026元/年，实现与国家扶贫标准有效衔接。协调推动医疗、医保、医药联动改革，医药卫生体制改革不断深化，全面取消公立医院药品加成，医德医风积极改善；卫生计生服务能力不断增强，新增床位670张，新增执业（助理）医师1527人，完成贫困村卫生室建设259个。完成农村适龄妇女“两癌”免费检查11万人。怀化科技馆、湖天群众文化展演中心、篮球综合馆项目进展顺利，老干活动中心建成使用，县级公共文体设施建设稳步推进。成功举办芷江、靖州30周年县庆活动，顺利举办市第五届运动会，成功承办全国柔道锦标赛。物价指数控制在1.6%以内，物价水平保持基本稳定。社会治理不断加强。社会治安综合治理民意调查排名全省第一，公安工作获评全国社会治安综合治理先进。扎实推进“七五”普法，社区矫正、安置帮教、人民调解工作水平稳步提升。依法管理宗教事务，宗教领域保持和谐稳定。金融秩序治理加强，清收压降不良贷款26.5亿元，其中公职人员不良贷款9180万元。侯卫东官场笔记全面开展“大排查大管控大整治”专项行动，安全生产及社会面火灾形势总体平稳。深入推进食品安全“四小”集中整治，切实加强药品监管，食品药品安全保障有力。其他事业取得新成效。奋力迎战历史罕见的洪涝灾害，紧急转移群众29.34万人，实现“零伤亡”。争取移民直补和后扶政策资金3.29亿元，完成特殊困难移民避险解困搬迁安置3542人。农村土地承包经营权确权登记颁证工作基本完成。村级“两委”换届选举圆满完成。全民国防教育、国防动员和人民防空工作扎实推进，预备役和民兵建设水平不断提升。工会、共青团、妇女儿童、科学普及等事业健康发展，审计、统计、档案、水文、气象、地方志、外事侨务、防震减灾、优抚安置、住房公积金等工作取得新成效。

政府自身建设得到加强。坚定不移全面从严治党，突出干事创业这个导向，政府各项工作规范有序。法治政府建设稳步推进。自觉接受人大法律监督、工作监督和政协民主监督。认真落实人大及其常委会的决议决定，办理人大代表建议235件；政协委员提案188件，答复率均达100%。贯彻中央全面依法治国部署要求，政府主要负责人履行法治建设第一责任人职责得到落实。严格履行重大行政决策法定程序，“三重一大”事项决策的科学性和民主性得到增强。政务环境不断优化。取消和下放行政审批事项212项，“互联网+政务服务”稳步推进。坚持常态化督查，狠抓懒政、怠政和为官不为等问题，不断完善绩效考核机制，行政效率进一步提升。正风肃纪成效显著。“两学一做”学习教育常态化制度化深入推进，“一岗双责”“两个责任”全面落实。从严查处侵害群众切身利益的问题，“雁过拔毛”式腐败、扶贫领域腐败和作风问题专项整治力度加大。持之以恒纠“四风”、治陋习，干部作风日趋好转。

各位代表，历尽天华成此景，人间万事出艰辛。过去的一年，发展成果令人鼓舞，所取成绩来之不易。这些成果和成绩的取得，是省委省政府和市委坚强领导的结果，是市人大市政协监督支持的结果，饱含了各位代表、各位委员的智慧和力量，凝聚了全市各族人民的辛勤和汗水。在此，我代表市人民政府，向全市广大干部群众，向各位代表、各位委员，向各民主党派、工商联、无党派人士和人民团体，向政法干警、驻怀解放军和武警、消防官兵，向中央、省驻怀单位，向离退休老同志，向所有关心支持怀化发展的各界朋友，表示衷心的感谢，并致以崇高的敬意！

在充分肯定成绩的同时，我们也清醒地认识到工作中仍存在一些问题与不足，主要是发展不平衡不充分的问题。一是经济总量不大、结构不优的问题依然存在；二是经济增长动力不强，实体经济尤其是企业生产经营困难仍然较多；三是地区生产总值、全社会固定资产投资等经济指标没有实现预期目标；四是产业发展短板仍然突出，产业投资占比不到30%，工业化率仅32.1%；五是脱贫攻坚任务依然十分艰巨，城乡差距依然较大；六是部分干部能力素质还需进一步提高，干部作风还需进一步改进等等。对这些问题和不足，我们一定高度重视，认真加以解决。

二、2018年工作安排

2018年，是贯彻党的十九大精神的开局之年，是改革开放40周年，是决胜全面建成小康社会、实施“十三五”规划承上启下的关键一年。做好全年工作，意义十分重大。

做好今年工作的总体要求是：全面贯彻党的十九大精神和中央、省委、市委经济工作会议精神，以习近平新时代中国特色社会主义思想为指导，加强党对经济工作的领导，坚持稳中求进工作总基调，坚持新发展理念，紧扣社会主要矛盾变化，按照高质量发展的要求，统筹推进“五位一体”总体布局和协调推进“四个全面”战略布局，坚持以供给侧结构性改革为主线，大力落实创新引领开放崛起战略，推进“三个着力”，突出发展产业、转型升级，奋力推进“一极两带”和“一个中心、四个怀化”建设，统筹推进稳增长、促改革、调结构、惠民生、防风险各项工作，在打好防范化解重大风险、精准脱贫、污染防治的攻坚战方面取得扎实进展，推动经济社会持续健康发展。

经济社会发展主要预期目标是：地区生产总值增长8.5%左右，规模工业增加值增长7.5%左右，全社会固定资产投资增长15%，社会消费品零售总额增长12%，一般公共预算收入增长10%，进出口总额增长40%，城、乡居民人均可支配收入分别增长8.5%、11%，居民消费价格涨幅控制在3%以内，万元GDP能耗下降3.2%。

围绕上述目标，必须突出抓重点、补短板、强弱项的工作方法，着力抓好五个方面工作。

（一）坚决打好三大攻坚战。按照十九大部署，重点打好防范化解重大风险、精准脱贫、污染防治“三大攻坚战”，使全面建成小康社会得到人民认可、经得起历史检验。

1.打好精准脱贫攻坚战。坚持以脱贫攻坚统揽经济社会发展全局，精细精准、用心用脑扎实推进脱贫攻坚工作，保质保量如期完成脱贫任务。严格落实脱真贫、真脱贫要求。实现12.8万贫困人口稳定脱贫，300个以上贫困村出列，辰溪、芷江、会同、靖州4县脱贫摘帽，完成易地扶贫搬迁2.1万人、农村危房改造1.9万户，完成25户100人以上自然村通水泥路3000公里、农村道路提质改造1200公里，巩固提升农村安全饮水24万人，完成贫困村农网改造升级254个、农村团寨消防安全改造160个。聚焦深度贫困精准发力。完善和落实“四跟四走”产业扶贫，全面推进“扶贫车间”进乡村，重点攻克2个深度贫困县和131个深度贫困村脱贫问题。激发贫困群众内生动力。注重扶贫同扶志、扶智相结合，推进教育扶贫、就业扶贫，引导贫困群众依靠自己脱贫致富。切实强化基层基础。扎实开展“三走访、三签字”活动，压紧压实市县乡村四级主体责任，切实强化脱贫攻坚实绩考核。巩固脱贫成果，做到脱贫不脱责任，脱贫不脱政策，脱贫不脱帮扶，脱贫不脱监管。强化贫困人口动态管理，切实抓好未脱贫人口和返贫人口的脱贫工作。

2.打好防范化解重大风险攻坚战。以建设“平安怀化”为目标，不断加强和创新社会治理，健全各方面风险防控机制，确保人民群众安居乐业。积极防范政府性债务风险。加快政府存量债务置换，实行政府债务限额管理，健全政府性债务风险预警、化解和应急处置机制。坚决制止违法违规举债融资行为，规范PPP项目运作，杜绝以保底承诺、回购安排、明股实债等方式变相融资。严格规范设立政府性基金，实现政府出资与社会资本同股同权、风险共担、收益共享。积极防范金融风险。集中整治银行贷款不良率高企问题、恶意逃废债行为，严厉打击非法集资行为，确保不发生系统性区域性风险。积极防范社会稳定风险。强化法治宣传教育，健全公共法律服务体系，发挥人民调解作用。创新和加强互联网治理，构建信息化社会治安立体防控体系。以信访工作法治年活动为主线，严格落实信访工作责任制，依法解决群众合理诉求。切实加强社会治安管理，严密防范和化解社会矛盾风险，高度重视和化解征地拆迁、环境保护、工资待遇等累积性风险。推动集中打击整治突出犯罪斗争深入开展，依法严厉打击黄赌毒、黑拐骗、枪抢盗等违法犯罪行为。积极防范安全风险。牢固树立安全生产红线意识，全面落实安全生产责任，持续推进交通、矿山、危险化学品、烟花爆竹、消防、建筑施工等重点行业领域专项整治，坚决遏制较大及以上事故发生。加强应急管理，提升综合防灾减灾救灾能力。大力实施食品药品安全战略，坚持严字当头，加强食品药品监管。

3.打好污染防治攻坚战。像保护眼睛一样保护生态环境，像对待生命一样对待生态环境，严守环境保护红线，营造天蓝水净地绿的美好家园。坚决打赢蓝天保卫战。大力开展城市大气污染整治专项行动，确保城区空气质量优良率稳定在90%以上，努力创建空气质量达标城市。切实加强水污染防治。全面推进河长制“3+X”专项整治行动，加强流域水环境治理，抓好城乡黑臭水体整治。完成中方县、辰溪县、洪江市等县市工业集中区污水处理设施建设，加快城东污水处理厂建设，完成怀化市全城污水处理厂、河西污水处理厂提质提标。加大饮用水水源保护，确保全市地表水考核断面水质达标率95%以上，集中式饮用水水源保护区水质达标率保持100%。突出抓好土壤污染防治。全面落实“土十条”，开展土壤污染治理与修复，坚决完成中央、省下达的重金属污染专项治理任务。持续加强生态系统保护。开展国土绿化行动，推进石漠化、水土流失综合治理，抓好湿地保护和恢复，强化地质灾害防治。严格落实耕地占补平衡制度，确保377万亩永久基本农田底线不突破。

（二）切实提高创新驱动能力和开放水平、大力发展产业振兴实体经济。按照高质量发展要求，全面贯彻创新引领开放崛起战略，不断增强经济创新力和竞争力。

1.扎实开展产业项目建设年活动。按照“产业项目建设年”要求，优先发展实体经济，启动产业及产业园区三年提升行动，积极对接省“五个100”行动，认真抓好10类重大产业项目，完成产业项目投资300亿元。着力发展优势产业。引导现代物流企业向怀化经开区等重点区域集聚，重点抓好佳惠武陵山农产品冷链园、传化智能公路物流港、义乌中国小商品城等重大项目，加快建设西南一流商贸物流基地。大力推进农旅、文旅融合发展，建设旅游商品研发推广中心，整合建设一批精品生态文化旅游线路、乡村旅游线路，新建一批高星级酒店和精品民宿、高等级景区、5星级农庄，不断完善旅游公共服务体系，强力实施旅游品牌营销，重点扶持旅游龙头企业发展，全力推进国家全域旅游示范市创建，实现全年接待游客5800万人次、旅游收入400亿元。以怀化高新区医药健康产业园、怀化经开区健康养老综合服务设施项目为龙头，以正清制药、博世康、补天药业、正好制药、新汇制药等企业为重点，积极培育医药健康产业链，加快五省边区医疗卫生健康服务中心建设步伐。发挥优质生态农产品资源丰富优势，依托怀化国家农业科技园，着力抓好农产品原材料供应基地、农产品加工基地、大宗农产品批发交易中心建设，加快建成全国绿色食品产业示范基地，积极培育大生物产业链。着力培育新兴产业。依托向华电子、奇力新电子、金升阳电子、伟兴鑫鑫等企业，加快发展电子信息产业。以怀化高新区水汽能生态能源智能制造项目、电动汽车充电设施研发项目、新晃县锦程新侗新能源汽车项目为重点，加快发展新能源和新能源汽车产业。以鹤城区远大住工、洪江市四维住工、辰溪润沅新材料、高新区骏泰新材料、洪江区久日新材等企业为重点，加快发展新材料产业。着力加快园区发展。以成功获批国家级高新区为契机，进一步理顺园区体制机制，加快园区基础设施和生产、生活性配套设施建设，完善园区服务功能，积极引导优势产业、企业向园区集聚，加快新型工业化步伐。怀化高新区重点发展生物医药、新材料、新能源、电子信息产业；怀化经开区重点发展现代服务业、高端制造业、外贸加工产业，争创国家级文化广告产业园；立足资源优势和产业基础，引导13个县市区工业集中区准确定位，实现错位发展。完成产业园区技工贸收入1400亿元，园区规模工业增加值达160亿元，工业集中度达65%。着力促进消费升级。巩固汽车等大宗消费，做大移动互联网等信息消费，拓展家政、健康、养老等新型消费。加快实体商业与互联网有效融合，实现电子商务交易额增长40%。办好汽车博览会、房地产交易会、健康产业博览会、农博会和生态文化旅游节等节会活动，促进会展经济快速发展。

2.深化供给侧结构性改革。全面落实“三去一降一补”重点任务，依法关闭不合规范的企业，加快房地产业转型升级，加大对实体经济的支持，切实减轻企业负担。统筹推进城市综合执法体制、农村集体产权制度、农村金融服务、医药卫生体制、投融资、财税、安全生产和国有企业等领域改革，加快全国集体林业综合改革试验示范区、经开区武陵山金融改革试验区建设，不断形成经济增长新动力。

3.切实加强创新体系和创新能力建设。围绕四大特色优势产业和战略性新兴产业发展，积极培育科技创新平台，加快科技成果转化，力争高新技术企业突破100家，完成全社会研发经费支出20亿元。深入推动“质量强市”战略，加快中国驰名商标、国际马德里商标和国家地理标志保护产品的培育力度。大力实施“五溪人才计划”，加大人才引进和培育力度，为产业发展提供强有力的智力支撑。

4.推动形成全方位开放格局。突出高位招商、亲情招商、以商招商、职能招商和驻点招商，发挥异地怀化商会作用，深入开展“对接湘商会、建设新家乡”活动，引进500强企业10家以上，力争所有县市区都有500强企业落户，全年实现招商引资到位资金700亿元。围绕高铁经济带建设，打通对外开放大通道，推进全方位高层次的区域经济合作。加快推动铁路口岸及综合保税物流中心报批建设。完成芷江机场航站楼改扩建，实现旅客吞吐量50万人次。开通怀新欧国际班列。实现怀邵衡铁路建成通车，加快张吉怀高铁、渝怀铁路增建二线、铁路编组站、动车运营所建设，实施焦柳铁路怀化段电气化改造，启动沅辰、官新、靖黎、芷铜等4条高速公路建设，力争怀芷高速建成通车，抓紧开展怀通桂（柳）高铁、雪峰山腹地旅游公路交通体系建设前期工作。继续实施外贸“破零倍增”行动，推动外企外资入怀和怀企怀品出境，支持云箭集团、向华电子、贝尔动漫等开拓国际市场，扩大外经外贸总量。

（三）着力加快城市提质步伐。抓紧启动国家卫生城市、国家森林城市、全国交通模范城市创建，扎实推进生态中心城市建设，让人民群众在城市生活得更方便、更舒心、更美好。

1.加快推进城市建设。坚持高起点城市规划，维护规划的法律性、严肃性、权威性，严格查违拆违，铁腕整治违建行为。继续推进高铁新城片区路网、城东组团路网基础设施、大湘西游客集散中心等项目建设；推进恒大、万达商业广场、步步高等新型城市综合体和新兴商圈建设；完成篮球综合馆、湖天群众文化展演中心建设；启动龙田湾特色文化街区建设；加快太平桥棚户区、月塘安置区、四方田城中村改造步伐；启动城市应急水源建设。

2.完善道路交通设施。实施红星路、正清路、锦溪路提质改造，加快四方路、香洲路、卢林南路等“断头路”建设，打通城市交通内循环；推进怀化城区大型综合停车场建设，新建停车场10个，新增停车位5000个；不断完善城市公交体系。

3.着力美化市容市貌。实施“厕所革命”三年行动计划，推动“厕所革命”从景区拓展到全域、从城市拓展到农村、从数量增加拓展到质量提升，年内主城区新建公厕30座，全市新建、改扩建旅游厕所103座；继续推进重点楼盘、重点地段、重点单位、重点楼体亮化工程；以全面实施《城市市容和环境卫生管理条例》为契机，运用智慧城管平台，以“绣花”功夫提升城市精细化管理水平，全力推进市容市貌、交通秩序、环境卫生等综合整治；扎实推进城市绿色化，抓好中坡国家森林公园景观绿化提质，启动湖天、迎丰公园二期、岩门等城市主题公园建设，新增街头小游园10个。

4.不断完善城镇体系。建成怀芷快速干线，启动建设怀洪旅游干线，实现鹤中洪芷生态城镇群快速通达。引导支持县城加快提质扩容步伐，高标准改造老城区，高品质建设新城区。继续抓好国家级、省级新型城镇化试点，着力推进沅陵县官庄镇、溆浦县北斗溪镇、鹤城区黄金坳镇、洪江市托口镇、通道县县溪镇等一批重点镇、特色镇建设。

（四）全面实施乡村振兴战略。坚持新型城镇化与乡村振兴两手抓两促进，把解决好“三农”问题摆在重中之重位置，按照产业兴旺、生态宜居、乡风文明、治理有效、生活富裕的总要求，努力实现农业农村优先发展。

1.加快发展现代农业。深化农业供给侧结构性改革，走质量兴农之路，推进农业由增产导向转向提质导向，加快构建现代农业产业体系、生产体系、经营体系，不断提高农业创新力、竞争力和全要素生产率。全面落实省三个“百千万”工程，重点扶持10个农业产业化龙头企业、100个农机专业合作社、1000个家庭农场。推进鹤城区九丰现代高效农业园、新晃县现代农业生态观光体验园等田园综合体建设，加快一二三产业融合发展。大力实施优质农产品品牌战略，重点推进碣滩茶、冰糖橙、杨梅、黄桃、葡萄、黄金贡柚等优质农产品基地建设。全面推动怀化国家农业科技园、靖州国家现代农业产业园、沅陵省级农业产业园提质升级。大力发展农产品加工业，全市农产品加工销售收入增长15%以上。

2.积极培育新型经营主体。支持农业骨干企业做大做强，重点开展家庭农场、农民专业合作社和星级休闲农庄示范创建，新增市级以上农业产业化龙头企业15家。鼓励返乡农民工、高校毕业生、科技人员投身现代农业建设，培育造就一支懂农业、爱农村、爱农民的“三农”工作队伍。加大农业技能培训力度，教育培训10万人次，积极培育新型职业农民。

3.大力推进美丽乡村建设。实施净化工程，重点抓好生活和医疗废物处理、生活污水治理和畜禽养殖污染治理等工作，切实加大农村环境综合整治。实施绿化工程，继续推进1000个“秀美村庄”建设，完成1000公里高速、高铁沿线两岸绿化提质和1000万亩封山育林，继续实施500万亩速生丰产林、100万亩优质油茶、100万亩高产楠竹基地建设，走乡村绿色发展之路，实现百姓富、生态美的统一。实施淳化工程，坚持物质文明和精神文明一起抓，加强农村公共文化建设，开展移风易俗行动，提升农民精神风貌，培育文明乡风、良好家风、淳朴民风。

（五）坚持在发展中保障和改善民生。牢固树立以人民为中心的发展思想，加快全面建成小康社会步伐，着力办好重点民生实事，着力补齐民生短板，不断增强群众获得感幸福感。

1.坚持教育优先发展。以省政府支持10所芙蓉学校建设为契机，加强教育基础设施建设，持续改善办学条件。重点完成实验中学、金时初中、碧桂园小学等学校建设，新增城区义务教育学位2万个，着力破解“大班额”问题。推进城乡义务教育一体化，确保中方、芷江、会同、沅陵通过国家义务教育均衡县评估验收。着力引进上海交通大学、中国农业大学等高校在怀化开放“专升本”招生。加强职业教育基础能力建设，完成芷江师范申创省幼儿师范高等专科学校。

2.扩大创业就业。抓好“创蚁”众创空间、高新区返乡创业园、武陵山大学生创客社区等创业平台建设，积极引导和支持各县市区产业园区建设创业孵化基地。加快“技能人才输送基地”共建步伐，提高就业质量和收入。新增城镇就业3.5万人，城镇登记失业率控制在4.5%以内，新增农村劳动力转移就业3.6万人。

3.完善社会保障体系。全面完成基金征缴任务，扩大社会保险覆盖面。完成省里下达的社会保障卡采集发行任务。巩固农村低保和社会保障兜底脱贫对象认定清理整顿成果，提高农村低保和兜底保障精准度。

4.提高健康服务水平。坚持预防为主，深入开展爱国卫生运动，倡导健康文明生活方式，预防控制重大疾病。强化医德医风建设，推进医联体建设，加快村卫生室建设，切实优化公共卫生服务。健全药品供应保障制度，减轻群众用药负担，有效缓解群众“看病贵”问题。完善计划生育服务管理，加强出生缺陷防治，提高出生人口素质。

5.推进文化事业发展。加快文化综合服务体系建设，加强文物保护利用和文化遗产保护传承，支持侗族村寨申世遗工作。支持麻阳办好自治县成立30周年庆典活动、会同县6个民族乡办好20周年乡庆活动，组织举办全市第五届少数民族传统体育运动会，推动5个民族自治县全面开展全国民族团结进步示范县创建活动。

同时，做好全国第四次经济普查工作；积极响应国防和军队改革，加强国防动员、民兵预备役、人民防空等工作，鼓励各类企业积极与军事院校、军工企业合作，促进民品军用和军品民用，推动军民融合发展；积极开展新一轮全省、全国双拥模范城创建工作；积极发展老龄事业和老年教育，扎实做好老干部工作；统筹做好民族宗教、外事侨务、审计、残联、档案、保密、气象、水文、移民、防震、地方志、住房公积金等各项工作。

三、政府自身建设主要任务

“打铁必须自身硬”，政府系统要突出政治建设统领地位，坚持以习近平新时代中国特色社会主义思想为指导，加强党对政府工作的领导，切实做到政治过硬、本领增强，努力建设法治政府、高效政府、服务型政府、创新政府和廉洁政府。

务必加强学习，做到绝对忠诚。要在学习中提升站位。坚持用党的十九大精神和习近平新时代中国特色社会主义思想武装头脑、指导实践、推动工作，牢固树立“四个意识”，坚定“四个自信”，坚决在思想上政治上行动上同以习近平同志为核心的党中央保持高度一致，坚决维护党中央权威和集中统一领导。要在学习中把准方向。坚决贯彻中央大政方针和省委市委决策部署，理清工作思路，突出工作重点，强化工作抓手，确保党对经济工作的领导落到实处。要在学习中创新思维。活学活用习近平新时代中国特色社会主义经济思想，努力提升战略思维、创新思维、辩证思维、法治思维、底线思维能力。

务必集思广益，做到科学决策。要大兴调查研究之风。深入基层、深入群众、深入一线，做到听真话、察实情、获真知，确保决策符合实际。要坚持问题导向。紧盯防范化解重大风险、精准脱贫、污染防治等重点领域，精准施策、有的放矢，确保决策务实有效。要扩大公众参与。建立公众参与平台，推行民意调查制度，公开决策信息，确保重大决策充分体现民意。

务必强化执行，做到落地生根。要雷厉风行不拖延。对市委的决策部署，必须做到不议论不争论不阔论，不折不扣抓落实。要强化“决策讲科学、办事讲程序、过程讲效率、结果讲圆满”的工作要求，强化“边说边干、少说多干、先干后说”的工作态度，强化“真抓实干、马上就办、办就办好”的工作作风，迅速将各项决策部署转化为具体行动、转化为发展实效。要久久为功不松劲。坚持一张蓝图绘到底，以钉钉子精神做细做实做好各项工作。对既定的重大战略、重大工作、重大项目要建立清单制度，强化督查督办，严格考核奖惩，坚决做到一以贯之、一鼓作气、一抓到底。要敢于担当不退缩。在产业发展中担重任、出新招，在深化改革中涉险滩、谋新路，在脱贫攻坚中打硬仗、创佳绩，努力解决表态多调门高、行动少落实差等突出问题。

务必尊崇法治，做到依法行政。要坚持依法履职。认真履行法治建设第一责任人职责，将政府工作全面纳入法治化轨道。加强政府法制机构建设，积极推行政府法律顾问和公职律师制度。要主动接受监督。依法接受人大及其常委会法律监督、工作监督，自觉接受人民政协的民主监督，认真办理人大代表建议和政协提案。主动接受人民群众和社会监督。虚心听取和采纳民主党派、工商联、无党派人士和人民团体的意见建议。要强化诚信意识。政府及其工作人员都要自觉做到言必信、行必果，说到做到。作出的决策，要定一条是一条，条条算数；部署的工作，要做一件成一件，件件落实；对群众和投资者作出的承诺，要说一项算一项，项项兑现。

务必创新管理，做到优质服务。要深化简政放权。进一步推动向园区赋权、向乡镇放权，继续精简市级行政审批项目，完善并联审批和联合审批运行机制，努力激发各类市场主体活力。要强化市场监管。坚决清理废除妨碍统一市场和公平竞争的各项规定和做法，营造公开公平公正的投资环境。要优化政务服务。整合提升各类服务平台，深入推进“最多跑一次”改革，加快智慧政务建设。扎实开展乱收费乱罚款乱摊派专项治理，大力引导支持民营经济发展，全面优化营商环境。

务必严守底线，做到廉洁自律。要始终知敬畏。时刻保持清醒头脑，坚持为民用权、谨慎用权、秉公用权，切实做到敬畏人民、敬畏权力、敬畏法律。要始终存戒惧。扎实开展“不忘初心、牢记使命”主题教育，坚定理想信念，自觉抵制各种不良倾向。强化团结协作意识，坚决做到不利于团结的话不说，不利于团结的事不做，努力营造相互补台、好戏连台的和谐氛围。要始终守底线。把严守政治纪律和政治规矩放在首要位置，严格落实党风廉政建设“一岗双责”，深入贯彻中央八项规定及省委九项规定精神，严格遵守“两准则、四条例”，驰而不息改作风、纠“四风”，构建亲清新型政商关系，确保干部清正、政府清廉、政治清明。

各位代表，怀化是我们的怀化，建设好怀化发展好怀化是我们共同的梦想和追求。这里有这么好的资源、这么好的优势、这么好的民风，我们没有理由不把工作干好。只要我们风雨同舟、齐心协力，我们的梦想就一定能实现！只要我们马上就办、真抓实干，我们的目标就一定能达到！只要我们坚持不懈、久久为功，怀化的明天就一定会更加美好！

各位代表，新时代，新征程，新作为，让我们紧密团结在以习近平同志为核心的党中央周围，在省委省政府和市委的坚强领导下，永不懈怠，一往无前，真抓实干，为加快建设“一极两带”和“一个中心、四个怀化”，实现怀化人民对美好生活的向往作出新的更大贡献！
